# Clinical predictors of late SARS‐CoV‐2 positivity in Italian internal medicine wards

**DOI:** 10.1111/eci.13705

**Published:** 2021-11-08

**Authors:** Federico Carbone, Stefano Ministrini, Sara Garbarino, Giulia Vischi, Valeria Carpaneto, Matteo Sobrero, Chiara Monti, Daria De Stefano, Benedetta Saccomanno, Marcella Massone, Luca Liberale, Arianna Piccardo, Alessandro Calvia, Federica Vischi, Maddalena Bagnasco, Ottavia Magnani, Matteo Caiti, Elisabetta Cenni, Paola Ballarino, Patrizia Giuntini, Alessandra Barreca, Chiara Tognoni, Federica Pirisi, Paolo Canepa, Domenico Cerminara, Lisa Pelanconi, Michele Strozzi, Amedeo Thneibat, Mario Stabile, Edineia Felix, Selena Dasso, Cecilia Casini, Alberto Minetti, Roberta Gonella, Fabio Ferrando, Andrea Bellodi, Alberto Ballestrero, Paolo Barbera, Andrea Lorenzo Poggi, Eleonora Arboscello, Aldo Pende, Paolo Moscatelli, Michele Piana, Fabrizio Montecucco

**Affiliations:** ^1^ Department of Internal Medicine First Clinic of Internal Medicine University of Genoa School of Medicine Genoa Italy; ^2^ IRCCS Ospedale Policlinico San Martino Genoa – Italian Cardiovascular Network Genoa Italy; ^3^ Internal Medicine Department "Santa Maria della Misericordia" Hospital University of Perugia Sant'Andrea delle Fratte Perugia Italy; ^4^ Center for Molecular Cardiology University of Zurich Schlieren Switzerland; ^5^ Dipartimento di Matematica LISCOMPLab University of Genoa Italy; ^6^ Clinica di Medicina d'Urgenza IRCCS Ospedale Policlinico San Martino Genoa Italy; ^7^ Divisione di Medicina d'Urgenza IRCCS Ospedale Policlinico San Martino Genoa Italy; ^8^ Department of Internal Medicine Clinic of Internal Medicine for Oncology University of Genoa School of Medicine Genoa Italy; ^9^ IRCCS Ospedale Policlinico San Martino Genoa Genoa Italy; ^10^ Emergency Department IRCCS Ospedale Policlinico San Martino Genoa Genoa Italy

**Keywords:** emergency department, ferritin, internal medicine, lactate dehydrogenase, mortality, SARS‐CoV‐2

## 1 INTRODUCTION

SARS‐CoV‐2 represents a “Pandora's box” highlighting critical issues for in‐hospital management of infectious patients. Over the past fifty years, the organization of hospital departments in Western countries has been driven by the need to face off noncommunicable diseases, in accordance with epidemiological data. Transmission‐based precautions have been instead limited to dedicated departments. During the last year, strong efforts have been then required to improve infection prevention strategies, a process involving countless aspects of patient management. In forerunners countries (e.g., Italy), a first step was to identify different in‐hospital paths for positive and nonpositive patients.^1^, ^2^ Due to the air contagiousness of disease, the identification and segregation of SARS‐CoV‐2‐positive patients from negative ones represented a critical step. As weeks went by, it became evident that the case definition of SARS‐CoV‐2 infection was not straightforward. Clinical features of SARS‐CoV‐2 infection (different from those observed in previous SARS and MERS pandemics),^3^ patients overcrowding and pitfalls in molecular diagnosis^4^ contributed together to overwhelm the traditional pre‐SARS‐CoV‐2 hospital organization. Clinicians had to lower the threshold of suspicion for deciding who needed to be tested/isolated and how to do that.^5^ When possible, separate pavilions were committed to SARS‐CoV‐2‐positive patients and somewhere for those presenting with high clinical suspicion of disease patients but testing negative for SARS‐CoV‐2 infection. Our Institution–IRCCS Policlinico San Martino–in Genoa (Italy) early approached such categorization of patients, being aware of the dramatic impact that an intrahospital spread would have. Even hospital facilities were then reorganised to segregate positive/suspicious patients to prevent the dissemination of the infection among SARS‐CoV‐2‐negative patients. By focussing on this critical point, we here retrospectively analyzed cases of late SARS‐CoV‐2 positivity occurring within internal medicine wards, formally not SARS‐CoV‐2‐dedicated. Clinical and management variables were investigated, aiming at identifying potential predictors of late SARS‐CoV‐2 positivity and their impact on patient outcome.^6^, ^7^ Alongside, potential value of common laboratory test at hospital admission was explored.

## 2 METHODS

### 2.1 Patient enrolment and assessment

This is a retrospective analysis of patients admitted to internal medicine wards not dedicated to SARS‐CoV‐2 between 24 February 2020 and 24 May 2020 at IRCCS Ospedale Policlinico San Martino in Genoa (Italy). The screened population was composed of 690 patients admitted during the enrolment period. Only those admitted from the ED and tested with at least one nasal‐pharyngeal swab for SARS‐CoV‐2 were included in the analysis (*n* = 668). Then, we excluded patients with positive for SARS‐CoV‐2 infection at ED reaching a final cohort of *n* = 478 (Figure S1). Clinical and biochemical data performed at ED admission have been collected from hospital records, including the provenance of patients (home, nursing home), any history of contact with SARS‐CoV‐2 cases and common symptoms of SARS‐CoV‐2 infection (i.e., fever, cough, dyspnoea, asthenia, anosmia, dysgeusia and diarrhoea). Comorbidities were then stratified according to the Charlson Comorbidity Index. Routinely, biochemical profile performed at ED admission and later during internal medicine ward hospitalization–including arterial blood gas analysis (BGA) and inflammatory biomarkers (i.e., fibrinogen, lactate dehydrogenase [LDH], ferritin and C‐reactive protein [CRP])–was retrieved from hospital records performed at ED admission–and later during internal medicine ward hospitalization–was retrieved from hospital records and included arterial blood gas analysis (BGA) and inflammatory biomarkers (i.e., fibrinogen, lactate dehydrogenase [LDH], ferritin and C‐reactive protein [CRP]). SARS‐CoV‐2 positivity of nasal‐pharyngeal swabs were performed by routine real‐time polymerase chain reaction. The present study was approved by the local ethics board of IRCCS Ospedale Policlinico San Martino (200/2020‐DB id 10515). The study was carried out in accordance with The Code of Ethics of the World Medical Association (Declaration of Helsinki) for experiments involving humans and adhered to the principles of the transparent reporting of a multivariable prediction model for individual prognosis or diagnosis (TRIPOD), the TRIPOD statement.

### 2.2 Study endpoints adjudication and sample size calculation

Cases of late positivity were defined as patients testing positive for SARS‐CoV‐2 in internal medicine wards, not SARS‐CoV‐2‐dedicated, after a previous negative molecular test performed in the ED (nasopharyngeal swab). The aim of the study was then to identify whether ED length of stay may have a role as predictor of late positivity to SARS‐CoV‐2 infection independently of other clinical variables, as emerging in literature.^7^, ^8^ They were selected among those significantly different at descriptive analyses: contact with suspected cases, fever and dyspnoea. Alongside, the same criteria were used to identify potential biochemical biomarkers: pCO2, fibrinogen, LDH, ferritin and CRP.

Sample size was calculated according to latest guidance for developing clinical prediction models.^9^ More specifically, by considering this study as a model with binary outcome, our sample size (*n* = 478) satisfied the minimum sample size required for (i) a 95% confidence interval for the overall outcome proportion of 0.5 (*n* ≥ 385); (ii) a mean absolute precision error (MAPE) <0.05 (*n* ≥ 252) and (iii) an expected uniform shrinkage factor <10% (*n* ≥ 340). Furthermore, we have preset a proportion of overall variance explained (*R* ^2^_cs_) = 0.1.

### 2.3 Statistical analysis

Analyses were performed with GraphPad Prism version 9.0.0 for Windows (GraphPad Software, San Diego, CA) and R environment for statistical computing (URL http://www. R‐ project. org/). Categorical data were presented as absolute and relative frequencies, whereas continuous ones as median and interquartile range [IQR] since the normality assumption was not demonstrated. Unpaired intergroup comparisons were drawn by Fisher's exact test and Mann‐Whitney *U*‐test, as appropriate. Logistic regression analyses were then used to estimate the predictive role toward a late nasal‐pharyngeal swab positivity to SARS‐CoV‐2 infection of clinical variables and biochemical parameters separately. Results were expressed as odds ratio (OR) with 95% confidence interval (CI). Model calibration performances have been evaluated via the Hosmer‐Lemeshow goodness of fit test with 10 groups. Model performance was assessed through receiver‐operator characteristic (ROC) curve analysis by reporting the area under the curve, sensitivity, specificity and +/− likelihood ratio. Predictive ability toward overall and SARS‐CoV‐2‐related mortality was tested through Cox hazard regression models presented as hazard ratio (HR) with 95% CI. Survival rate was also estimated with Kaplan‐Meier curve and log‐rank test. For all statistical analyses, a 2‐sided *p*‐value <0.05 was considered as statistically significant. Based on a minimum measurable difference of 1 day between the groups in our primary endpoint, a late positivity group >25 patients implies a statistical power >95%, as calculated with the two‐tailed Mann‐Whitney *U*‐test.

## 3 RESULTS

### 3.1 Longer stay in emergency department is associated with late positivity for SARS‐CoV‐2 infection

Clinical characteristics of the study cohort are reported in Table S1. Patients were almost all Caucasian (99.0%), elderly (median age of 80 years [70–86]) and equally distributed across sexes (231 males, 48.3%). When study cohort was categorised in persistent negative vs. late SARS‐CoV‐2‐positive, the latter group was associated with history of previous contact with suspicious or ascertained cases (24.5% vs. 5.8%, *p* = 0.003) and clinical presentation with fever (65.0% vs. 25.6%, *p* < 0.001) and dyspnoea (62.5% vs. 25.3%, *p* < 0.001) at admission. Except for the history of ischemic stroke and peptic ulcer disease (Table S2), the impact of comorbidity burden–as assessed by Charlson Comorbidity Index–on late SARS‐CoV‐2 positivity was not significant. Conversely, even a slightly longer stay at ED was significantly associated with late SARS‐CoV‐2 positivity (3 vs. 2 days, *p* = 0.012). Since patients with late SARS‐CoV‐2 positivity were immediately transferred to dedicated units, the median length of stay in internal medicine wards, not SARS‐CoV‐2‐dedicated, was lower in this group of patients (7 days^3^, ^4^, ^5^, ^6^, ^7^, ^8^, ^9^, ^10^, ^11^, ^12^, ^13^ vs. 10 days,^6^, ^7^, ^8^, ^9^, ^10^, ^11^, ^12^, ^13^, ^14^, ^15^, ^16^, ^17^ *p* < 0.001), without significant differences in the overall hospitalization time (Table S1).

At BGA, only pCO2 was slightly lower in late SARS‐CoV‐2‐positive patients (33 mmHg vs. 35 mmHg, *p* = 0.040) (Table S3), whereas biochemical profile showed an inflamed status characterised by increased serum levels of fibrinogen (*p* = 0.029), LDH (*p* = 0.038), ferritin (*p* = 0.002) and CRP (*p* = 0.002).

### 3.2 Longer stay at emergency department predicts late SARS‐CoV‐2 positivity

**FIGURE 1:**
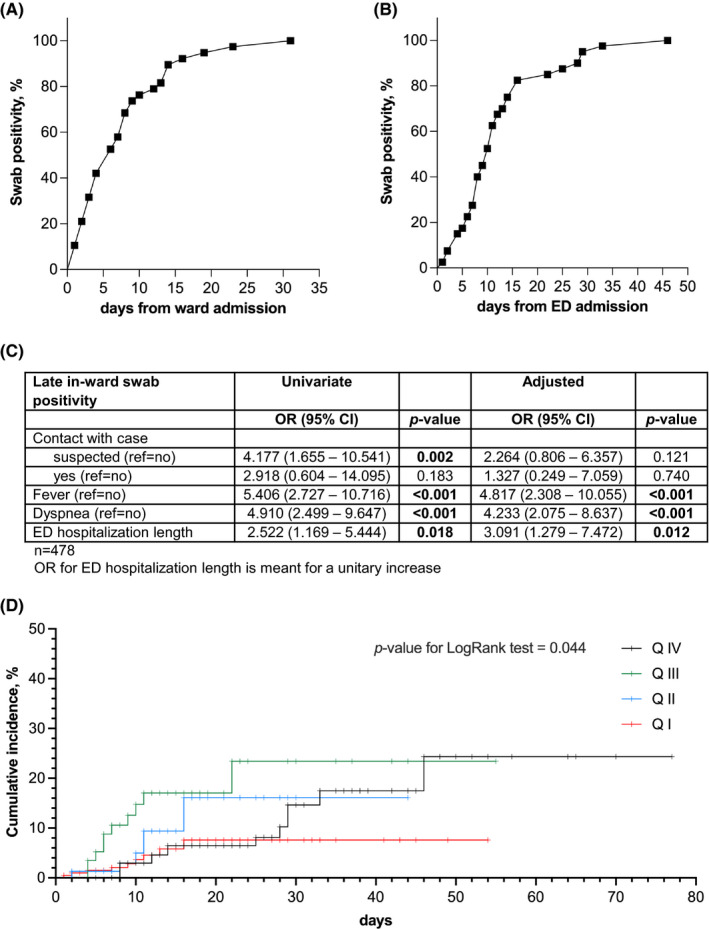
Clinical and biochemical factors influence late SARS‐CoV‐2 positivity in patient admitted in internal medicine ward. Cumulative SARS‐CoV‐2 positivity occurs early after hospital admission: (A) cumulative incidence of positivity after admission to internal medicine wards, not dedicated to SARS‐CoV‐2 (data points represent the day the patients became positive); (B) cumulative incidence of positivity after admission to emergency department (ED) (data points represent the day the patients became positive). Alongside with in‐ED length of stay, clinical and management variables are independently associated with late SARS‐CoV‐2 positivity (C) and even a slightly longer ED permanence is associated with an increased cumulative risk (D). QI‐QIV: quartiles of length of stay in ED

Out of 478 hospitalizations in internal medicine wards not SARS‐CoV‐2‐dedicated, a total of 40 patients presented late positivity (8.3%). Positivity generally occurred within 14 days from in‐ward admission (92.5%, Figure 1A) and ED admission (75.0% Figure 1B). When included in logistic regression model, fever (OR 5.406 [2.727–10.716]: *p* < 0.001), dyspnoea (OR 4.910 [2.499–9.647]: *p* < 0.001) and ED hospitalization length (OR 3.091 [1.279–7.472]: *p* = 0.012) emerged as clinical predictors of late SARS‐CoV‐2 positivity at nasal‐pharyngeal swab (Figure 1C). Combining those variables allowed to improve the predictive performance–especially the sensitivity–as emerging from ROC curve analysis (Figure S2). We have then internally validated our results by performing a bootstrap resampling performance. Based on 1000 bootstrap replicates, we obtained the following estimate of the ORs (average of the 1000 ORs from the 1000 bootstrap samples) and related 95% CI. Noteworthy, the OR estimated on the original dataset falls within the new bootstrap confidence intervals (Figure S3). Conversely, no inflammatory biomarkers showed an independent association with late SARS‐CoV‐2 positivity (Table S4). Further emphasising the predictive role of even a slightly longer ED hospitalization, the Kaplan‐Meier analysis showed that risk of late SARS‐CoV‐2 substantially increased according to quartiles of length of stay in ED (p‐value for log‐rank test 0.044, Figure 1D).

### 3.3 Longer stay at emergency department predicts overall in‐hospital mortality during the first wave SARS‐CoV‐2 pandemic

Patients discharge after hospitalization in internal medicine wards formally not SARS‐CoV‐2‐dedicated took place mostly at home (60.88%), while frailer patients were transferred to nursing homes (5.8%) (Table S5). Among late SARS‐CoV‐2‐positive patients, 75.0% required hospitalization in SARS‐CoV‐2‐dedicated wards, whereas only 10% was discharged at home. Overall, mortality rate during the study period was 12.6% (*n* = 60), whereas only 2 late SARS‐CoV‐2‐positive patients died. Clinical variables associated with late SARS‐CoV‐2 positivity were then included in a Cox proportional regression model in which only ED hospitalization length was confirmed as the only predictor of overall in‐hospital mortality (HR 1.029 [1.005–1.054]; *p* = 0.018]) (Table 1). When biochemical variables were considered, both LDH (adjusted HR 5.219 [4.251–10.125]; *p* = 0.022) and ferritin (adjusted HR 1.747 [1.082–2.822]; *p* = 0.022) were confirmed as independent predictors of overall in‐hospital mortality (Table S6).

**TABLE 1:** Univariate Cox regression model including potential clinical predictors for in‐hospital mortality

Overall mortality	Univariate	*p*‐value
	HR (95% CI)	
Contact with case		
Suspected (ref = no)	1.613 (0.949–2.741)	0.077
Yes (ref = no)	0.646 (0.206–2.027)	0.454
Fever (ref = no)	1.313 (0.959–1.798)	0.090
Dyspnoea (ref = no)	1.258 (0.913–1.732)	0.160
History of TIA/stroke (ref = no)	1.323 (0.882–1.985)	0.176
History of peptic ulcer disease (ref = no)	1.403 (0.520–3.785)	0.503
ED hospitalization length	1.029 (1.005–1.054)	**0.018**

Note

Abbreviations: CI, Confidence interval; ED, Emergency department; HR, Hazard ratio; TIA, Transitory ischemic attack.

## 4 DISCUSSION

The present study has been designed to address a challenging clinical problem emerged during the first wave of SARS‐CoV‐2 pandemic. Alongside restrictive measures instituted by the governments, hospitals had to reorganise themselves–each in its own way–according to their characteristics: location, pavilion architecture, epidemiology and available medical staff among the others.^10^ Here, we highlighted a critical role for length of ED stay as predictor of late positivity within internal medicine wards not SARS‐CoV‐2‐dedicated. Although we considered a 14 days window as suggested by the WHO, the median incubation time for SARS‐CoV‐2 infection was six days long, in line with reported in literature.^11^, ^12^ Being the median ED hospitalization length shorter (three days), both out‐hospital infection and in‐hospital spreading of SARS‐CoV‐2 infection should be considered. Interestingly, the strongest independent predictors were fever and dyspnoea, typical presentation symptoms of SARS‐CoV‐2 infection. It would be then conceivable to suppose that at least a part of positive patients were initially false negatives. This hypothesis implies these cases to be more likely out‐hospital rather than in‐hospital infections. However, Figure S4 clearly indicates that late SARS‐CoV‐2‐positive patients were tested several times before positivity occurred (in median 4 times) and often with different type of tests. This observation supports the role of in‐hospital infection. Being this the only partially modifiable risk factor among those included in the model, it represents a clinical challenge of undoubted clinical relevance. This aspect has been widely described even in the early phase of pandemic, when positive patients attending small EDs in the North of Italy spread the infection to working personnel, other patients and relatives.^10^ SARS‐CoV‐2 infection, indeed, remained unrecognised for several weeks in Italy–and Europe as well–and no special measures were taken to reduce the spread in the early phase of contagion. This accounts for a large part of patients admitted to internal medicine wards without any molecular screening test for SARS‐CoV‐2 infection (and then excluded from our analysis). Most of them were admitted to ED before or in the early phase of pandemic (December 2019‐February 2020). The other patients admitted without SARS‐CoV‐2 screening were those considered negative because of clinical presentation. Those behaviours have progressively changed as clinical pitfalls in SARS‐CoV‐2 infection diagnosis have been demonstrated. Furthermore, ED admittance of patients with nondeferrable disease also resumed as the weeks went by (March‐April 2020), alongside with SARS‐CoV‐2 patients with less severe–or even atypical–disease presentation. Here, the need to identify and segregate patients at high, intermediate and low risk of carrying SARS‐CoV‐2 dramatically emerged.^13^, ^14^ Floor‐to‐ceiling partitions were built to cordon off part of the main ED. Moreover, risk of exposure for supposedly negative patients also had to be guaranteed during transport towards radiology or other facilities. As regional emergency hub, our institution then had to maintain an active surveillance of local pandemic evolution dynamically reassessing workflow processes not only in the ED but also in the whole hospital.^15^, ^16^ As a result, late SARS‐CoV‐2 positivity occurred in 8.3% of patients admitted in internal medicine wards not SARS‐CoV‐2‐dedicated. Noteworthy, ED length of stay significantly impacts on overall in‐hospital mortality. Keep some internal medicine wards free of SARS‐CoV‐2 infection should indeed represent a key achievement for preventing hospital collapse during SARS‐CoV‐2 pandemic. Maintaining patient outflow from ED, preserving standard of care for patients and diseases not SARS‐CoV‐2‐related, reducing contagion risk after discharge: these main benefits are worth keeping some internal medicine wards free from SARS‐CoV‐2 infection. Some may argue that over 1.5 years into the pandemic, those key aspects have already been implemented. However, it should be considered that the coming fall‐winter season will be likely the first where SARS‐CoV‐2 and seasonal influenza viruses will coexist. Vaccination campaigns have indeed significantly reduced the population at risk of SARS‐CoV‐2 infection, but they also resulted in a softening of containment measures. An upcoming stress for ED is then expectable due to a paradoxical increased risk of contagion and to the challenges in terms of differential diagnosis between SARS‐CoV‐2 and influenza viruses. Keep surveillance on what concerns patient triage, segregation and outflow will therefore represent challenges to be addressed with the lessons of pandemic. Furthermore, this experience showed how inconsistent the pandemic plans were worldwide. Any *post*‐*hoc* analysis may then provide a contribution for their redesigning/implementation, although healthcare organization greatly differs at several levels between countries – and even regions, cities and hospitals within. This is a clinical study that shows a special focus on ED length of stay. On the one hand, this aspect might hinder the reproducibility of the study results in other countries especially considering potential differences in ED length of stay. Despite our attempt of internally validate our results, larger studies are warranted to perform multivariable analyses and extensive predictive modelling.

Another aspect that should be considered is the diagnostic performance of the combined nasal and throat swab, which is characterised by high specificity but limited sensitivity.^17^ As repeated testing significantly improved diagnostic performance, this may represent a bias that should be addressed in larger studies.

In conclusion, this retrospective analysis found that the duration of stay at the ED before the admission to ordinary wards represents a potential risk factor for late in‐hospital positivity to SARS‐CoV‐2 diagnostic tests. Despite the implementation of prevention protocols, the high variability in virus incubation time might determine a failure of preventive measures. A dynamic reassessment of workflow processes throughout the hospital then represents a critical step for preventing hospital collapse during SARS‐CoV‐2 pandemic. Institution of quarantine sites within each clinical ward (as done at IRCCS San Martino Hospital in Genoa) might be more helpful to manage the risk of late positivity. Finally, also laboratory variables (i.e., ferritin and LDH) may help, with some limitations, in predicting late SARS‐CoV‐2 positivity and mortality.

## CONFLICT OF INTEREST

The authors report no relationships that could be construed as a conflict of interest.

## Supporting information

Supplementary Material

## Data Availability

Datasets are available on request by the Corresponding Author.

## References

[1] Carbone F , Montecucco F . SARS‐CoV‐2 outbreak and lockdown in a Northern Italy hospital. comparison with Scandinavian no‐lockdown country. Eur J Clin Invest. 2020:e13302. 32506507 10.1111/eci.13302 PMC7300436

[2] Santi L , Golinelli D , Tampieri A , et al. Non‐COVID‐19 patients in times of pandemic: emergency department visits, hospitalizations and cause‐specific mortality in Northern Italy. PLoS One. 2021;16:e0248995. 33750990 10.1371/journal.pone.0248995 PMC7984614

[3] Cevik M , Tate M , Lloyd O , Maraolo AE , Schafers J , Ho A . SARS‐CoV‐2, SARS‐CoV, and MERS‐CoV viral load dynamics, duration of viral shedding, and infectiousness: a systematic review and meta‐analysis. Lancet Microbe. 2021;2:e13‐e22. 33521734 10.1016/S2666-5247(20)30172-5 PMC7837230

[4] Premraj A , Aleyas AG , Nautiyal B , Rasool TJ . Nucleic Acid and Immunological Diagnostics for SARS‐CoV‐2: processes, platforms and pitfalls. Diagnostics (Basel). 2020;10:866. 10.3390/diagnostics10110866 PMC7690661 33114057

[5] Wee LE , Fua TP , Chua YY , et al. Containing COVID‐19 in the emergency department: the role of improved case detection and segregation of suspect cases. Acad Emerg Med. 2020;27:379‐387. 32281231 10.1111/acem.13984 PMC7262126

[6] Lumley SF , Constantinides B , Sanderson N , et al. Epidemiological data and genome sequencing reveals that nosocomial transmission of SARS‐CoV‐2 is underestimated and mostly mediated by a small number of highly infectious individuals. J Infect. 2021;83(4):473‐482. 34332019 10.1016/j.jinf.2021.07.034 PMC8316632

[7] Pung R , Lin B , Maurer‐Stroh S , et al. Factors influencing SARS‐CoV‐2 transmission and outbreak control measures in densely populated settings. Sci Rep. 2021;11:15297. 34315928 10.1038/s41598-021-94463-3 PMC8316572

[8] Nam NH , Tien PTM , Truong LV , et al. Early centralized isolation strategy for all confirmed cases of COVID‐19 remains a core intervention to disrupt the pandemic spreading significantly. PLoS One. 2021;16:e0254012. 34264966 10.1371/journal.pone.0254012 PMC8282022

[9] Riley RD , Ensor J , Snell KIE , et al. Calculating the sample size required for developing a clinical prediction model. BMJ. 2020;368, m441. 32188600 10.1136/bmj.m441

[10] Perico N , Fagiuoli S , Di Marco F , et al. Bergamo and Covid‐19: how the dark can turn to light. Front Med (Lausanne). 2021;8:609440. 33681246 10.3389/fmed.2021.609440 PMC7933506

[11] Yu P , Zhu J , Zhang Z , Han Y . A familial cluster of infection associated with the 2019 novel coronavirus indicating possible person‐to‐person transmission during the incubation period. J Infect Dis. 2020;221:1757‐1761. 32067043 10.1093/infdis/jiaa077 PMC7107453

[12] Lauer SA , Grantz KH , Bi Q , et al. The incubation period of coronavirus disease 2019 (COVID‐19) from publicly reported confirmed cases: estimation and application. Ann Intern Med. 2020;172:577‐582. 32150748 10.7326/M20-0504 PMC7081172

[13] Tan RMR , Ong GY , Chong SL , Ganapathy S , Tyebally A , Lee KP . Dynamic adaptation to COVID‐19 in a Singapore paediatric emergency department. Emerg Med J. 2020;37:252‐254. 32321705 10.1136/emermed-2020-209634

[14] Yaffee AQ , Peacock E , Seitz R , et al. Preparedness, adaptation, and innovation: approach to the COVID‐19 pandemic at a decentralized, quaternary care department of emergency medicine. West J Emerg Med. 2020;21:63‐70. 33052812 10.5811/westjem.2020.8.48624 PMC7673894

[15] Gamberini L , Coniglio C , Cilloni N , et al. Remodelling of a regional emergency hub in response to the COVID‐19 outbreak in emilia‐romagna. Emerg Med J. 2021;38(4):308‐314. 33574025 10.1136/emermed-2020-209671

[16] Manauis CM , Loh M , Lim AHJ , et al. The next wave: key adaptations to operational workflows of national screening centre (Singapore) and the emergency department during the COVID‐19 pandemic. Int J Emerg Med. 2021;14:14. 33627063 10.1186/s12245-021-00337-w PMC7903370

[17] Lee KK , Doudesis D , Ross DA , et al. Diagnostic performance of the combined nasal and throat swab in patients admitted to hospital with suspected COVID‐19. BMC Infect Dis. 2021;21:318. 33823800 10.1186/s12879-021-05976-1 PMC8022129

