# Supplementary material for: Clinical predictors of late SARS‐CoV‐2 positivity in Italian internal medicine wards
Source: Eur J Clin Invest. 2021 Nov 8;52(1):e13705. doi: 10.1111/eci.13705 (PMC8646747; doi:10.1111/eci.13705)
Supplement: Supplementary file 1 — Supplementary Material [file ECI-52-0-s001.doc]

# Supplementary Material

# Figure S1. Flow diagram illustrating patient selection

**
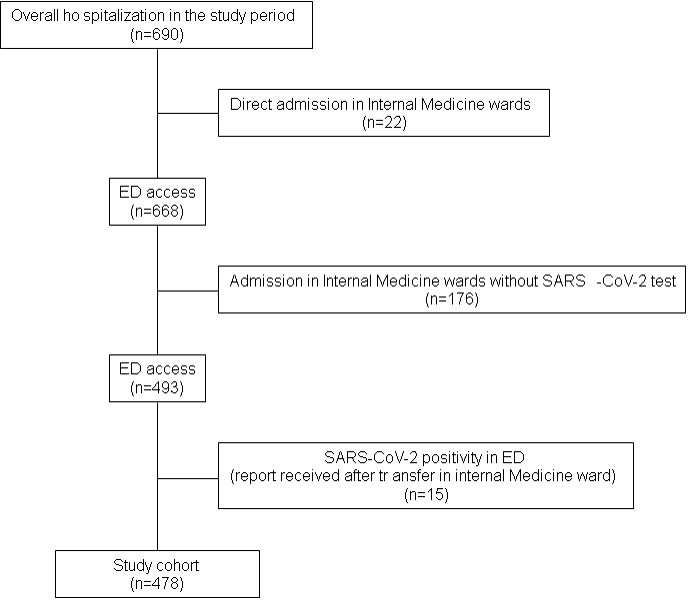
**

# Table S1. Clinical and anthropometric parameters of patients admitted at Emergency Department and then transferred to Internal Medicine wards after negative nasopharyngeal swab.

|  | **Overall**  **(n=478)** | **Persistent negative**  **(n=438)** | **Late positive**  **(n=40)** | ***p*-value** |
| --- | --- | --- | --- | --- |
| Age, years [IQR] | 80 [70 – 86] | 80 [70 – 86] | 80 [75 – 86] | 0.511 |
| Sex, male (%) | 231 (48.3) | 207 (47.3) | 24 (60.0) | 0.138 |
| Post-menopausal women, yes (% among women) | 231 (93.5) | 216 (89.6) | 15 (93.8) | 0.969 |
| Ethnicity |  |  |  | 0.794 |
| Caucasian, n (%) | 473 (99.0) | 433 (98.9) | 40 (100.0) |  |
| North Africans, n (%) | 4 (0.9) | 4 (0.9) | 0 (0.0) |  |
| Sub-Saharans Africans, n (%) | 0 (0.0) | 0 (0.0) | 0 (0.0) |  |
| Hispanic, n (%) | 1 (0.2) | 1 (0.2) | 0 (0.0) |  |
| Other, n (%) | 0 (0.0) | 0 (0.0) | 0 (0.0) |  |
| Origin |  |  |  | 0.235 |
| Home, n (%) | 438 (91.6) | 407 (92.9) | 31 (77.5) |  |
| Senior residence, n (%) | 29 (6.1) | 22 (5.0) | 7 (17.5) |  |
| Other, n (%) | 11 (2.3) | 9 (2.1) | 2 (5.0) |  |
| Contact with case |  |  |  | **0.003** |
| None, n (%) | 428 (91.8) | 38 (94.1) | 43 (75.4) |  |
| Suspected, n (%) | 27 (5.8) | 19 (4.6) | 8 (14.0) |  |
| Yes, n (%) | 11 (2.4) | 5 (1.2) | 6 (10.5) |  |
| Fever, yes (%) | 138 (28.9) | 112 (25.6) | 26 (65.0) | **<0.001** |
| Cough, yes (%) | 14 (2.9) | 12 (2.7) | 2 (5.0) | 0.330 |
| Dyspnea, yes (%) | 136 (28.5) | 111 (25.3) | 25 (62.5) | **<0.001** |
| Asthenia, yes (%) | 133 (27.8) | 125 (28.5) | 8 (20.0) | 0.275 |
| Anosmia, yes (%) | 74 (15.5) | 72 (16.4) | 2 (5.0) | 0.066 |
| Dysgeusia, yes (%) | 2 (0.4) | 1 (0.2) | 1 (2.5) | 0.161 |
| Diarrhea, yes (%) | 15 (3.1) | 12 (2.7) | 3 (7.5) | 0.122 |
| Smokers |  |  |  | 0.102 |
| None, n (%) | 291 (61.1) | 265 (60.8) | 26 (65.0) |  |
| Previous, n (%) | 123 (25.8) | 110 (25.2) | 13 (32.5) |  |
| Yes, n (%) | 62 (13.0) | 61 (14.0) | 1 (2.5) |  |
| Hypertension, yes (%) | 292 (61.3) | 268 (61.5) | 24 (60.0) | 0.867 |
| Charlson comorbidity index [IQR] | 7 [5 – 9] | 7 [5 – 9] | 7 [5 – 10] | 0.689 |
| Weight, Kg [IQR] | 70 [60 – 80] | 70 [60 – 80] | 70 [60 – 80] | 0.870 |
| Body temperature, C° [IQR] | 36.5 [36.2 – 37.2] | 36.5 [36.2 – 37.1] | 36.8 [36.3 – 37.8] | **0.043** |
| Respiratory frequency, acts/min [IQR] | 20 [18 – 24] | 18 [16 – 22] | 20 [14 – 24] | 0.588 |
| Heart rate, bpm [IQR] | 86 [75 – 100] | 86 [76 – 100] | 84 [74 – 110] | 0.860 |
| sBP, mmHg [IQR] | 130 [118 – 150] | 130 [118 – 150] | 133 [115 – 150] | 0.977 |
| dBP, mmHg [IQR] | 75 [65 – 85] | 75 [65-85] | 80 [60 – 80] | 0.785 |
| ED hospitalization length, days [IQR] | 2 [1 – 4] | 2 [1 – 4] | 3 [2 – 6] | **0.012** |
| In-ward hospitalization length, days [IQR] | 10 [6 – 17] | 10 [6 – 17] | 7 [3 – 13] | **0.001** |
| Overall hospitalization length, days [IQR] | 13 [9 – 22] | 13 [9 – 22] | 13 [8 – 19] | 0.215 |

Continuous data are presented as median [interquartile range] whereas categorical ones as absolute (relative) count. The *p*-values refers to the comparison between persistent negative and late positive patients (Mann-Whitney test or Fisher exact test, as appropriate).

sBP: systolic blood pressure; dBP: diastolic blood pressure; ED: emergency department.

# Table S2. Comorbidities at admission according with the Charlson comorbidity index.

|  | **Overall**  **(n=478)** | **Persistent negative**  **(n=438)** | **Late positive**  **(n=40)** | ***p*-value** |
| --- | --- | --- | --- | --- |
| History of myocardial infarction, yes (%) | 95 (20.1) | 87 (20.1) | 8 (20.0) | 1.000 |
| Chronic heart failure, yes (%) | 140 (29.5) | 127 (29.2) | 13 (32.5) | 0.717 |
| Peripheral vascular disease, yes (%) | 76 (16.1) | 68 (15.7) | 8 (20.0) | 0.499 |
| History of TIA/stroke, yes (%) | 64 (13.5) | 54 (12.4) | 10 (25.0) | **0.048** |
| Dementia, yes (%) | 76 (16.0) | 69 (15.8) | 7 (17.5) | 0.821 |
| COPD, yes (%) | 98 (20.6) | 91 (20.9) | 7 (17.5) | 0.688 |
| Connective tissue disease, yes (%) | 17 (3.6) | 16 (3.7) | 1 (2.5) | 1.000 |
| History of peptic ulcer disease, yes (%) | 9 (1.9) | 6 (1.4) | 3 (7.5) | **0.033** |
| Liver disease |  |  |  | 0.540 |
| None, n (%) | 438 (92.2) | 399 (91.7) | 39 (97.5) |  |
| Mild, n (%) | 16 (3.4) | 15 (3.4) | 1 (2.5) |  |
| Moderate, n (%) | 11 (2.3) | 11 (2.5) | 0 (0.0) |  |
| Severe, n (%) | 10 (2.1) | 10 (2.3) | 0 (0.0) |  |
| Diabetes |  |  |  | 0.153 |
| None or under diet control, n (%) | 353 (74.6) | 320 (73.9) | 33 (82.5) |  |
| Uncomplicated (%) | 87 (18.4) | 84 (19.4) | 3 (7.5) |  |
| End-organ damage, n (% | 33 (7.0) | 29 (6.7) | 4 (10.0) |  |
| Hemiplegia, yes (%) | 10 (2.1) | 9 (2.1) | 1 (2.5) | 0.589 |
| Moderate-to-severe CKD, yes (%) | 110 (23.3) | 102 (23.6) | 8 (20.5) | 0.843 |
| Solid malignancy, yes (%) | 132 (27.8) | 118 (27.1) | 14 (35.0) | 0.356 |
| Leukemia, yes (%) | 10 (2.1) | 8 (1.8) | 2 (5.0) | 0.202 |
| Lymphoma, yes (%) | 14 (2.9) | 13 (3.0) | 1 (2.5) | 1.000 |
| AIDS, yes (%) | 1 (0.2) | 0 (0.0) | 1 (2.5) | 0.084 |
| Charlson comorbidity index [IQR] | 7 [5-9] | 7 [5-9] | 6 [5-10] | 0.689 |

Data are presented as absolute (relative) count. Comparisons were drawn by Fisher exact test.

TIA: transient ischemic attack; COPD: chronic obstructive pulmonary disease; CKD: chronic kidney disease; AIDS: acquired immunodeficiency syndrome.

# Table S3. Laboratory findings characterizing patients admitted at Emergency Department.

|  | **Overall**  **(n=478)** | **Persistent negative**  **(n=438)** | **Late positive**  **(n=40)** | ***p*-value** |
| --- | --- | --- | --- | --- |
| SaO2, % [IQR] | 95 [90 – 97] | 95 [90 – 97] | 95 [91 – 97] | 0.964 |
| pO2, mmHg [IQR] | 70 [58 – 80] | 70 [57 – 80] | 72 [59-83] | 0.711 |
| P/F [IQR] | 326 [269 – 374] | 326 [267 – 374] | 318 [281 – 384] | 0.751 |
| pCO2, mmHg [IQR] | 35 [32 – 40] | 35 [32 – 40] | 33 [30 – 36] | **0.040** |
| HCO3^-^ [IQR] | 25 [22 – 28] | 25 [22 – 28] | 25 [23 – 28] | 0.866 |
| Lactates [IQR] | 1.3 [0.9 – 2.1] | 1.3 [0.9 – 2.1] | 1.5 [0.8 – 2.3] | 0.838 |
| Hb, g/dL [IQR] | 12.1 [10.3 – 13.8] | 12.1 [10.3 – 13.8] | 11.7 [9.7 – 13.4] | 0.428 |
| WBC, cells x 10^3^/mm3 [IQR] | 9.7 [6.9 – 12.6] | 9.6 [6.8 – 12.4] | 10.8 [7.0 – 15.4] | 0.312 |
| Neutrophil count, cells/mm3 [IQR] | 7.5 [5.0 – 10.3] | 7.4 [5.0 – 9.9] | 8.7 [4.8 – 13.5] | 0.183 |
| Platelets, cells x 10^3^/mm3 [IQR] | 235 [169 – 318] | 235 [169 – 318] | 259 [161 – 322] | 0.868 |
| PT, % [IQR] | 75 [62 – 86] | 76 [62 – 87] | 68 [61 – 79] | 0.067 |
| INR [IQR] | 1.23 [1.11 – 1.42] | 1.22 [1.11 – 1.42] | 1.32 [1.18 – 1.43] | 0.131 |
| aPTT, sec [IQR] | 32 [29 – 36] | 32 [29 – 36] | 33 [29 – 36] | 0.680 |
| D-dimer, ng/mL [IQR] | 1643 [984 – 4960] | 1640 [975 – 5076] | 1855 [1054 – 4303] | 0.889 |
| Fibrinogen g/L [IQR] | 4.52 [3.20 – 6.10] | 4.43 [3.01 – 6.46] | 5.98 [3.76 – 6.46] | **0.029** |
| Creatinine, mg/dL [IQR] | 1.1 [0.8 – 1.6] | 1.1 [0.8 – 1.6] | 1.2 [0.8 – 1.6] | 0.634 |
| eGFR, mL/min [IQR] | 59 [35 – 80] | 60 [34 – 80] | 50 [38 – 80] | 0.627 |
| Total bilirubin, mg/dL [IQR] | 0.60 [0.42 – 0.89] | 0.61 [0.43 – 0.94] | 0.50 [0.38 – 0.79] | 0.112 |
| LDH, U/L [IQR] | 235 [185 – 326] | 232 [183 – 318] | 284 [211 – 377] | **0.038** |
| Ferritin, ng/mL [IQR] | 179 [57 – 458] | 142 [50 – 425] | 420 [189 – 933] | **0.002** |
| CRP, mg/L [IQR] | 30 [7 – 89] | 26 [6 – 83] | 62 [26 – 129] | **0.002** |
| IL-6, pg/mL [IQR]) | 198 [73 – 632] | 196 [64 – 585] | 240 [119 – 2078] | 0.494 |

Data are presented as median [interquartile range]. The *p*-values refers to the comparison between persistent negative and late positive patients (Mann-Whitney test or Fisher exact test, as appropriate).

SaO2. O2 saturation, pO2: pressures of oxygen; P/F: pressure of oxygen/fractional inspired oxygen ratio; pO2: pressures of carbon dioxide; HCO3^-^: bicarbonate; Hb: hemoglobin, WBC: white blood cells; PT: prothrombin time; INR: international normalized ratio; aPTT: activated partial thromboplastin time; eGFR: estimated glomerular filtration rate; LDH: Lactate dehydrogenase; CRP: C-reactive protein; IL: interleukin. The *p*-values refers to the comparison between persistent negative and late positive patients (Mann-Whitney test).

# Figure S2. Model performance for late in-ward swab positivity assessed by receiver operator characteristic (ROC) curve analysis.

**
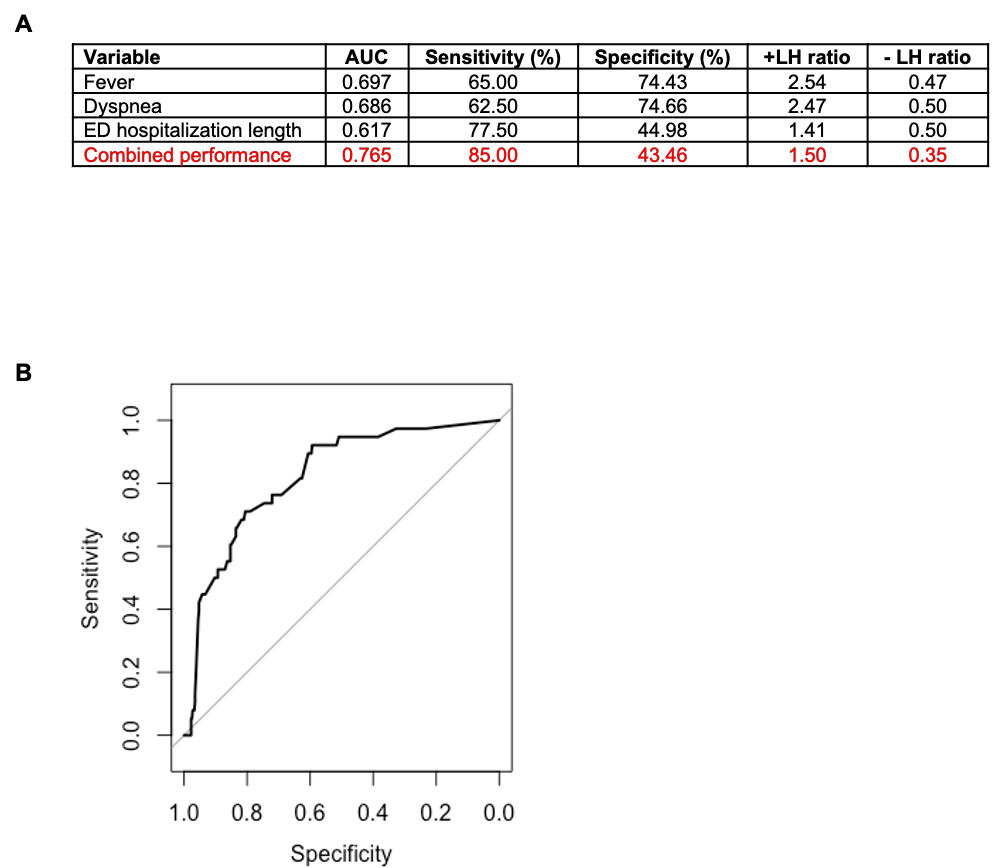
**

ROC curve analysis show model performance (**A**). Panel **B** provide image of ROC curve for the model combining fever, dyspnea and emergency department (ED) hospitalization length. AUC: area under the curve; LH: likelihood; ED: emergency department

# Figure S3. Internal validation of prediction model for late in-ward swab positivity through bootstrap resampling performance


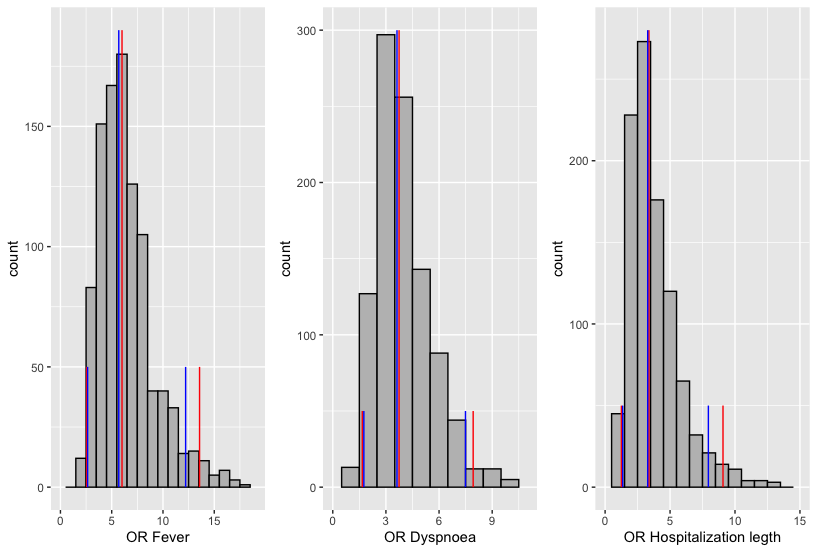


Panels show the distribution of the 1000 ORs from the 1000 bootstrap samples for the 3 predictors of interest. In blue the estimated OR and their 95% CI of the original dataset, and in red the average estimated OR and their 95%CI on the bootstrap samples.

# Table S4. Logistic regression model including potential biochemical predictors for late in-ward swab positivity at admission in Emergency Department.

| **Late in-ward swab positivity** | **Univariate** |  | **Adjusted** |  |
| --- | --- | --- | --- | --- |
|  | **OR (95% CI)** | ***p*-value** | **OR (95% CI)** | ***p*-value** |
| pCO2 | 0.938 (0.871 – 1.009) | 0.085 |  |  |
| Fibrinogen | 14.508 (1.203 – 174.935) | **0.035** | 2.701 (0.052 – 140.152) | 0.622 |
| LDH | 4.310 (1.019 – 18.219) | **0.047** | 13.834 (0.720 – 265.919) | 0.082 |
| Ferritin | 3.421 (1.476 – 7.926) | **0.004** | 1.376 (0.405 – 4.678) | 0.610 |
| CRP | 2.510 (1.405 – 4.484) | **0.002** | 2.012 (0.387 – 10.453) | 0.406 |

OR: relative risk; CI: confidence interval; LDH: lactate dehydrogenase; CRP: C-reactive protein

# Table S5. Descriptive analysis of discharge among the study cohort.

|  | **Overall**  **(n=478)** | **Late positive**  **(n=40)** |
| --- | --- | --- |
| Home, n (%) | 289 (60.5) | 4 (10.0 |
| Nursing home n (%) | 28 (5.9) | 0 (0.0) |
| In-hospital quarantine n (%) | 29 (6.1) | 24 (60.0) |
| ICU n (%) | 4 (0.8) | 3 (7.5) |
| Deceased n (%) | 60 (12.6) | 2 (5.0) |
| Other, n (%) | 68 (14.2) | 7 (17.5) |

ICU: intensive care unit.

# Table S6. Univariate Cox regression model including potential biochemical predictors for in-hospital mortality.

| **Overall mortality** | **Univariate** | | **Adjusted** | |
| --- | --- | --- | --- | --- |
|  | **HR (95% CI)** | **p-value** | **HR (95% CI)** | ***p*-value** |
| pCO2 | 1.001 (0.998 – 1.014) | 0.887 |  |  |
| Fibrinogen | 0.504 (0.146 – 1.744) | 0.280 |  |  |
| LDH | 5.219 (2.540 – 10.725) | **<0.001** | 4.251 (1.785 – 10.125) | **0.001** |
| Ferritin | 2.137 (1.448 – 3.154) | **<0.001** | 1.656 (1.046 – 2.622) | **0.031** |
| CRP | 2.014 (1.564 – 2.595) | **<0.001** | 1.562 (0.944 – 2.586) | 0.083 |

HR: hazard ratio; CI: confidence interval; LDH: lactate dehydrogenase; CRP: C-reactive protein.

# Figure S4. Case series reporting number and type of SARS-CoV-2 tests in the subgroup of late positive patients performed in both Emergency department and Internal Medicine wards.


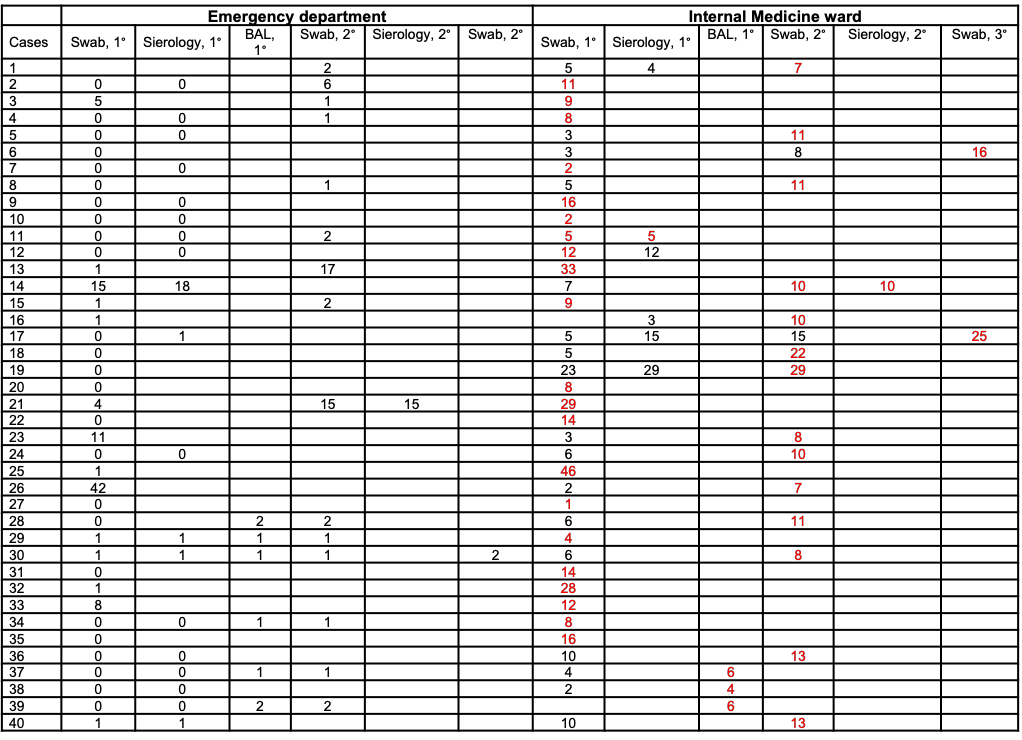


Progressive numbers (column cases) indicate late positive patients. Numbers in the other columns indicate the days from admission in Emergency Department. In red test positive for SARS-CoV-2 infection. The median number of tests for each patient is 4. BAL: bronchoalveolar lavage

| **Section/Topic** | **Item** | **Checklist Item** | **Page** |
| --- | --- | --- | --- |
| **Title and abstract** | | | |
| Title | 1 | Identify the study as developing and/or validating a multivariable prediction model, the target population, and the outcome to be predicted. | 1 |
| Abstract | 2 | Provide a summary of objectives, study design, setting, participants, sample size, predictors, outcome, statistical analysis, results, and conclusions. | N/A |
| **Introduction** | | | |
| Background and objectives | 3a | Explain the medical context (including whether diagnostic or prognostic) and rationale for developing or size validating the multivariable prediction model, including references to existing models. | 3 |
|  | 3b | Specify the objectives, including whether the study describes the development or validation of the model or both. | 3 |
| **Methods** | | | |
| Source of data | 4a | Describe the study design or source of data (e.g., randomized trial, cohort, or registry data), separately for the development and validation data sets, if applicable. | 3 |
|  | 4b | Specify the key study dates, including start of accrual; end of accrual; and, if applicable, end of follow-up. | 3 |
| Participants | 5a | Specify key elements of the study setting (e.g., primary care, secondary care, general population) including number and location of centres. | 3 |
|  | 5b | Describe eligibility criteria for participants. | 3 |
|  | 5c | Give details of treatments received, if relevant. | N/A |
| Outcome | 6a | Clearly define the outcome that is predicted by the prediction model, including how and when assessed. | 4 |
|  | 6b | Report any actions to blind assessment of the outcome to be predicted. | N/A |
| Predictors | 7a | Clearly define all predictors used in developing or validating the multivariable prediction model, including how and when they were measured. | 4 |
|  | 7b | Report any actions to blind assessment of predictors for the outcome and other predictors. | N/A |
| Sample size | 8 | Explain how the study size was arrived at. | 4-5 |
| Missing data | 9 | Describe how missing data were handled (e.g., complete-case analysis, single imputation, multiple imputation) with details of any imputation method. | N/A |
| Statistical analysis methods | 10a | Describe how predictors were handled in the analyses. | 4-5 |
|  | 10b | Specify type of model, all model-building procedures (including any predictor selection), and method for internal validation. | 4-5 |
|  | 10d | Specify all measures used to assess model performance and, if relevant, to compare multiple models. | 4-5 |
| Risk groups | 11 | Provide details on how risk groups were created, if done. | 4-5 |
| **Results** | | | |
| Participants | 13a | Describe the flow of participants through the study, including the number of participants with and without the outcome and, if applicable, a summary of the follow-up time. A diagram may be helpful. | 3 &  Suppl. Fig. 1 |
|  | 13b | Describe the characteristics of the participants (basic demographics, clinical features, available predictors), including the number of participants with missing data for predictors and outcome. | Suppl. Table 1 |
| Model development | 14a | Specify the number of participants and outcome events in each analysis. | Fig 1, Table 1, Suppl. Tables 1-6 |
|  | 14b | If done, report the unadjusted association between each candidate predictor and outcome. | Fig. 1, Table 1, Suppl. Tables 1-6 |
| Model specification | 15a | Present the full prediction model to allow predictions for individuals (i.e., all regression coefficients, and model intercept or baseline survival at a given time point). | N/A |
|  | 15b | Explain how to the use the prediction model. | N/A |
| Model performance | 16 | Report performance measures (with CIs) for the prediction model. | Figure S2 |
| **Discussion** | | | |
| Limitations | 18 | Discuss any limitations of the study (such as nonrepresentative sample, few events per predictor, missing data). | 9 |
| Interpretation | 19b | Give an overall interpretation of the results, considering objectives, limitations, and results from similar studies, and other relevant evidence. | 10 |
| Implications | 20 | Discuss the potential clinical use of the model and implications for future research. | 10 |
| **Other information** | | | |
| Supplementary information | 21 | Provide information about the availability of supplementary resources, such as study protocol, Web calculator, and data sets. | 10 |
| Funding | 22 | Give the source of funding and the role of the funders for the present study. | 10 |

We recommend using the TRIPOD Checklist in conjunction with the TRIPOD Explanation and Elaboration document.
